# Low Birth Weight, β-Cell Function and Insulin Resistance in Adults: The Brazilian Longitudinal Study of Adult Health

**DOI:** 10.3389/fendo.2022.842233

**Published:** 2022-03-14

**Authors:** Julia Ines F. Branda, Bianca de Almeida-Pititto, Isabela Bensenor, Paulo A. Lotufo, Sandra Roberta G. Ferreira

**Affiliations:** ^1^ Department of Epidemiology, School of Public Health, University of São Paulo, São Paulo, Brazil; ^2^ Center of Clinical and Epidemiological Research at University of São Paulo, São Paulo, Brazil; ^3^ Department of Preventive Medicine, Federal University of São Paulo, São Paulo, Brazil; ^4^ Department of Internal Medicine, Medical School, University of São Paulo, São Paulo, Brazil

**Keywords:** low birth weight, early life events, beta cell function, insulin sensitivity, prediabetes

## Abstract

**Background:**

Adverse intrauterine environment—reflected by low birth weight (LBW)—has been linked to insulin resistance and type 2 diabetes later in life. Whether β-cell function reduction and insulin resistance could be detected even in middle-aged adults without overt diabetes is less investigated. We examined the association of LBW with β-cell function and insulin sensitivity in non-diabetic middle-aged adults from the Brazilian Longitudinal Study of Adult Health (ELSA-Brasil).

**Methods:**

This is a cross-sectional analysis of 2,634 ELSA-Brasil participants aged between 34 and 59 years, without diabetes. Participants were stratified according to LBW defined as <2.5 kg and their clinical data were compared. HOMA-IR, HOMA-β, HOMA-adiponectin, TyG index, QUICKI and TG/HDL were calculated and their association with LBW were tested using multiple linear regression including adjustments suggested by Directed Acyclic Graphs and propensity score matching was applied.

**Results:**

The sample (47.4 ± 6.3 years) was composed of 57.5% of women and 9% had LBW. Subjects with LBW and normal-weight reported similar BMI values at the age of 20 years and current BMI was slightly lower in the LBW group. In average, cardiometabolic risk profile and also indexes of β-cell function and insulin sensitivity were within normal ranges. In regression analysis, log-transformed HOMA-β—but not with the other indexes—was associated with LBW (p = 0.014) independent of sex, skin color, prematurity, and family history of diabetes. After applying propensity-score matching in a well-balanced sample, HOMA-AD and TG/HDL indexes were associated with LBW.

**Conclusion:**

The association between LBW and insulin sensitivity markers may occur in healthy middle-aged adults before overt glucose metabolism disturbances. Our data are coherent with the detection of early life events consequent with insulin resistance markers that could contribute to the risk of glucose metabolism disturbances.

## Introduction

Diabetes mellitus remains one of the most relevant public health concerns worldwide due to its micro and macrovascular complications (1). The etiology of type 2 diabetes mellitus (T2DM) is multifactorial, involving genetic, environmental, and lifestyle factors (2) and is commonly accompanied by excess body adiposity.

Based on the Developmental Origins of Health and Disease (DOHaD) theory, cardiometabolic disorders in adulthood might have their origins early in life stemming from intrauterine insults, namely, maternal and fetal undernutrition (3), maternal smoking, alcohol consumption or health conditions in perinatal life (4). As an adaptation to survive under adverse gestational conditions, fetal programming occurs, resulting in structural and functional changes in body organs and systems (5, 6).

Low birth weight (LBW), a proxy of intrauterine adversity, has been associated with adult-onset diseases, namely, obesity, T2DM and the metabolic syndrome (7, 8). It has been reported that LBW is associated with decreased β-cell mass and reduced function, resulting in a low insulin response to glucose levels (9, 10). Additional underlying mechanisms have been related to evidence of decreased insulin sensitivity in the genesis of glucose metabolism disturbance, concomitant with progressive β-cell dysfunction during adulthood (11, 12). Studies show that perinatal stress may affect insulin action in peripheral organs with reduced glucose uptake, and decreased expression of GLUT4 glucose transport by muscle and adipose cells (13–15). This condition becomes particularly worrisome considering the tendency of weight gain associated with our current environment and lifestyle. In this context, greater awareness of glucose metabolism abnormalities is important for early identification of risk later in adult life.

A number of studies have associated LBW with T2DM (16, 17), although identifying an association of birth weight with impaired insulin sensitivity and β-cell function before the onset of diabetes, the focus of interest of the present study, would be more opportune for preventive measures.

The Brazilian Longitudinal Study of Adult Health (ELSA-Brasil) is a large cohort study of adult health in Brazil, designed to investigate risk factors associated with diabetes and cardiovascular disease (18, 19). Therefore, the ELSA-Brasil represents an opportunity to investigate associations of early life events with outcomes in adulthood. The present study examined the association of LBW with parameters of β-cell function and insulin sensitivity in non-diabetic participants of the ELSA-Brasil.

## Methods

### Study Design and Population

A cross-sectional analysis was carried out of baseline data from the multicenter ELSA-Brasil study, whose methodological details have been reported elsewhere (18, 19). The baseline assessment was conducted from August 2008 to December 2010 and included 15,105 employees aged 35–74 years from six Brazilian universities and research institutions. The present analysis drew on the baseline data of 5,061 participants of both sexes, aged 35–74 years from the São Paulo center. The study was approved by the local Ethics Committee and informed consent was obtained from all participants.

### Eligibility Criteria

**Figure 1:**
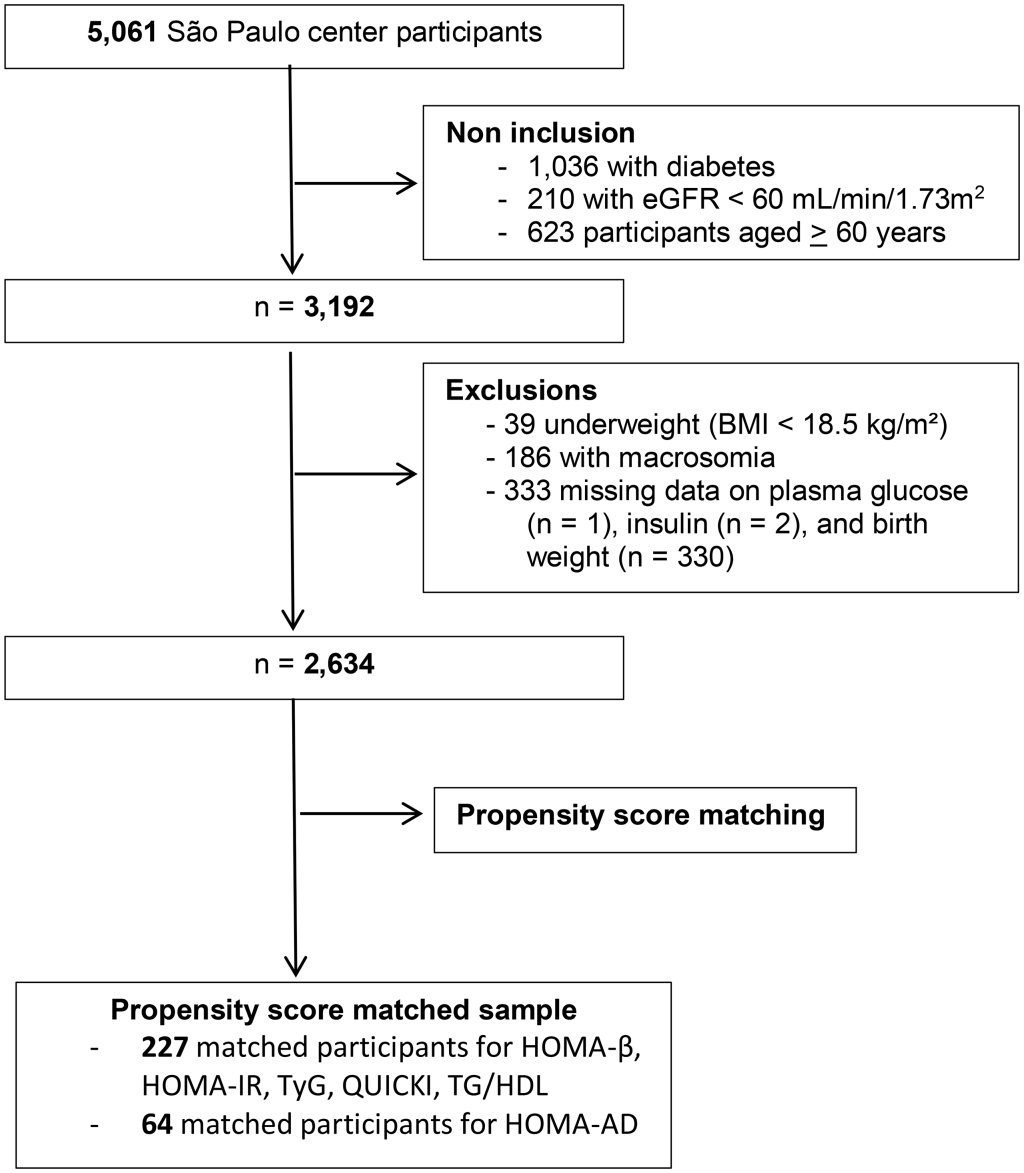
Flowchart of selection of ELSA-Brasil participants for study inclusion.

To be eligible for the present study, participants had to be aged <60 years (to reduce recall bias), non-diabetic and have preserved renal function. Of the 5,061 participants, the following subjects were excluded: 1,036 with diabetes (self-reported or in use of antidiabetic medications or newly diagnosed), 210 with glomerular filtration rate <60 mL/min/1.73 m^2^ or macroproteinuria, and 623 aged ≥60 years (20). Thirty-nine participants were subsequently excluded for being underweight (BMI <18.5 kg/m^2^) and 186 because they were born with macrosomia (birth weight >4.0 kg). A further 333 participants were excluded for missing data on exposure (birth weight) or outcome (plasma glucose, insulin and lipids) variables. Therefore, a total of 2,634 participants were included in the present study (**Figure 1**).

### Clinical and Laboratory Data

Participants were interviewed using standardized questionnaires (21). Self-reported data regarding demographics, socioeconomic status and health conditions were obtained. Variables of interest were age (years), sex (male, female), skin color (black, white, brown, yellow or indigenous, further stratified into white and non-white categories), family history of diabetes and hypertension (yes or no) and maternal educational level of participant.

Prematurity (yes or no) and birth weight (kg) were self-reported when possible. Birth weight was also categorized into “<2.5 kg”, “2.5–4.0 kg”, “>4.0 kg” or “unknown”. All participants were also asked to provide their body weight at 20 years of age.

Weight and height were measured and body mass index (BMI) then calculated as weight in kilograms divided by height in meters squared to express nutritional status. Waist circumference was measured at the midpoint between the last rib and the iliac crest using an inelastic tape. Blood pressure was measured using an Omron HEM 705CPINT device (Omron Co, Kyoto, Japan) after a 5-minute rest in a sitting position. Three measurements were taken at 1-min intervals and mean values calculated. After overnight fasting, blood samples were collected and participants then underwent a 2-hour 75 g oral glucose tolerance test. Fasting and 2-hour plasma glucose and insulin were determined. Aliquots were frozen at −80°C for further determinations (22, 23).

Plasma glucose was measured by the hexokinase method (ADVIA Chemistry; Siemens, Deerfield, Illinois, USA), and glycated hemoglobin determined by high-pressure liquid chromatography (Bio-Rad Laboratories, Hercules, California, USA) according to the National Glycohemoglobin Standardization Program certified method. Insulin (Siemens, Tarrytown, USA) and adiponectin (Enzo Life Sciences, Farmingdale, NY, USA) were determined using enzyme-linked immunoenzymatic assays.

The HOMA-β and HOMA-IR indexes were used to assess β-cell function and insulin sensitivity, respectively, and were calculated using the equations:


HOMA-β=[20×fasting insulin (µUI/ml)]/[fasting glucose (mmol/L)−3.5]

HOMA-IR=[fasting insulin (µUI/ml)×fasting glucose (mmol/L)]/22.5

Additionally, insulin sensitivity was evaluated by HOMA-adiponectin (HOMA-AD), the Triglycerides–glucose index (TyG index), QUICKI (Quantitative Insulin Sensitivity Check Index) and the Triglyceride-to-HDL-c ratio (TG/HDL-c), using the following equations: HOMA-AD = fasting glucose (mmol/L) × fasting insulin (mU/L)/22.5 × adiponectin (µg/ml); TyG index = log [(fasting triglycerides (mg/dl) × fasting glucose (mg/dl)]/2; QUICKI = 1/(log insulin (µUI/ml) + (log fasting glucose (mg/dl) and TG/HDL-c.

Adiponectin was measured in a sub-sample of 1,000 participants. After applying exclusion criteria, 742 participants were included with available adiponectin data.

Total cholesterol was assessed using the enzymatic colorimetric method (ADVIA Chemistry; Siemens, Deerfield, Illinois, USA). HDL-c was determined by the homogeneous colorimetric method without precipitation, and triglycerides by the glycerophosphate peroxidase method according to the Trinder assay (ADVIA Chemistry; Siemens, Deerfield, Illinois, USA). LDL-c concentrations were calculated using the Friedewald equation.

### Definitions for Analyses

Birth weight (exposure variable) was classified into three categories: low birth weight (<2.5 kg), normal birth weight (2.5–4.0 kg) and macrosomia (>4.0 kg). Prematurity was defined by an affirmative answer to the question: “*Were you a premature baby, in other words, were you born earlier than expected*?”. Outcomes were HOMA-β, HOMA-IR, HOMA-AD, TyG index, QUICKI and TG/HDL-c, analyzed as continuous variables.

Nutritional status was classified into underweight (BMI <18.5 kg/m^2^), normal weight (18.5–24.9 kg/m^2^), overweight (25.0–29.9 kg/m^2^) and obesity (>30.0 kg/m^2^). Hypertension was diagnosed when systolic or diastolic blood pressure levels were ≥140 or 90 mmHg, respectively, or when participant was in use of antihypertensive drugs.

Diabetes was diagnosed according to the American Diabetes Association criteria (24), as follows: fasting plasma glucose ≥126 mg/dl or 2-hour post challenge >200 mg/dl or glycated hemoglobin ≥6.5%. Prediabetes (yes or no) was defined as fasting plasma glucose between 100 and 125 mg/dl or 2-hour post challenge between 140 and 199 mg/dl or glycated hemoglobin 5.7–6.4%.

### Statistical Analysis

Distribution normality was tested for continuous variables and those with non-normal distribution (HOMA-β, HOMA-IR, HOMA-AD, QUICKI, and TG/HDL-c) were log-transformed before analysis to achieve normality. Continuous variables with a normal distribution were expressed as mean ± standard deviation (SD) and compared using Student´s *t*-test. Non-normally distributed variables were expressed as median and interquartile range and compared using the Wilcoxon rank test. Categorical variables were expressed as absolute and relative frequencies and compared by the chi-squared test.

Associations of exposure (LBW) and outcome (HOMA-β, HOMA-IR, HOMA-AD, TyG index, QUICKI, and TG/HDL-c) variables were initially analyzed by simple linear regression. Directed Acyclic Graphs (DAG) were used to build theoretical models and analyze independent associations of exposure with outcomes in multiple linear regression analyses. The DAG is a causal diagram which allows scientific evidence regarding the relationships among variables to be incorporated in graphics software to reach the ideal set of covariables (minimum sufficient adjustment) for the model to prevent biases and overadjustments (25, 26). Figures were created by DAGitty software, version 3.0 (www.dagitty.net) included in the **Supplementary Material** (**Figures S1A, B**).

Based on the DAGs, the association of LBW with HOMA-β and parameters of insulin sensitivity were adjusted for sex, skin color, family history of diabetes, and prematurity.

Considering the difference in sample size between groups with normal birth weight and LBW, and potential selection bias due to the nature of the study, propensity score matching was employed to create more comparable groups (27, 28). The nearest neighbor-matching algorithm within a caliper of 0.1 SD of logit function of propensity score was used. First, for the propensity score matching, a multiple logistic regression model was used, adjusted for DAG-based covariates (sex, skin color, family history of diabetes, and prematurity), and the probability of each participant having LBW *versus* normal birth weight was estimated. Balance between the groups was assessed by comparing each covariate. When standardized mean difference fell in the −0.1 to 0.1 range, groups were considered balanced. This matching reduced all covariate imbalance in the sample. The matched sample was then submitted to multiple linear regression in order to analyze associations of LBW (exposure as independent variable) with β-cell function and insulin sensitivity markers (outcomes as dependent variables) adjusted for the same DAG-based covariates. Tests were performed using “MatchIt”, “rbounds”, “Matching”, “twang” and “survey” packages in the R statistical environment.

All analyses were performed using the R Project for Statistical Computing software (R version 3.5.2) and statistical significance was set at a p-value of 0.05.

## Results

For the study sample of 2,634 participants, mean age was 47.4 ± 6.3 years, 57.5% were women and 59.3% reported white skin color. In general, the cardiometabolic parameters of the sample were within normal ranges (systolic and diastolic blood pressures of 116.6 ± 14.9 and 74.2 ± 10.4 mmHg, respectively), except for overweight (26.8 ± 4.6 kg/m^2^) and prediabetic (plasma glucose of 102.0 ± 7.7 mg/dl) status.

A total of 238 (9.0%) participants reported LBW and 145 (5.5%) were born preterm. LBW participants were predominantly women (61.7%), had white skin color (52.2%), and reported low maternal educational level (62.0%).

Participants with LBW had a higher rate of low educational level compared to those reporting normal birth weight (16.1% *versus* 10.8%, p = 0.007). LBW and normal-weight groups reported similar BMI values at the age of 20 years and current BMI was slightly lower in the LBW group, with borderline significance (p = 0.075, **Table 1**). Mean values of waist circumference were higher in the normal birth weight than the LBW group (88.2 ± 11.6 *versus* 86.0 ± 11.7 cm, p = 0.008), but both values were, on average, within normal ranges. Blood pressure levels and lipid metabolism variables were similar for the two groups. No differences in beta-cell secretion and insulin sensitivity indexes were found between the groups.

**Table 1:** Clinical characteristics of participants born with normal and low birth weight.

	Normal Birth Weight	Low Birth Weight	*P-*value
	n = 2,582	n = 238	
Age (years)	47.4 (6.3)	47.5 (6.4)	0.657
Body mass index at age 20 (kg/m^2^)	21.8 (3.4)	20.9 (3.0)	0.125
Body mass index (kg/m^2^)	26.9 (4.6)	26.3 (4.5)	0.075
Waist circumference (cm)	88.2 (11.6)	86.0 (11.7)	0.008
Systolic blood pressure (mmHg)	116.5 (14.9)	117.4 (15.3)	0.417
Diastolic blood pressure (mmHg)	74.2 (10.4)	74.7 (10.6)	0.507
LDL-cholesterol (mg/dl)	129.7 (33.0)	132.5 (33.1)	0.224
HDL-cholesterol (mg/dl)	56.7 (14.4)	57.0 (13.4)	0.328
Triglycerides (mg/dl)	130.2 (91.9)	126.5 (71.3)	0.449
Fasting plasma glucose (mg/dl)	102.6 (8.4)	102.7 (7.9)	0.832
Glycated hemoglobin (%)	5.2 (0.54)	5.3 (0.57)	0.963
Fasting insulin (mg/dl)	6.0 (3.5–10.0)	5.9 (3.2–8.9)	0.181
2-h plasma glucose (mg/dl)	122.0 (24.0)	122.9 (27.5)	0.610
2-h insulinemia	43 (26.7–69.1)	41.2 (26.0–64.6)	0.434
HOMA-IR*	2.5 (1.66–3.5)	2.4 (1.58–3.4)	0.187
HOMA-β*	56.0 (32.9–91.9)	53.9 (30.8–83.8)	0.189
HOMA-AD*	0.43 (0.22–0.96)	0.42 (0.19–1.10)	0.776
TyG	2.0 (0.12)	2.1 (0.11)	0.959
QUICKI*	0.36 (0.33–0.39)	0.37 (0.34–0.40)	0.186
TG/HDL*	1.9 (1.3–3.2)	2.0 (1.4–2.9)	0.824

HOMA-IR, Homeostasis Model Assessment of Insulin Resistance; AD, Adiponectin; TyG, Triglycerides glucose index; QUICKI, Quantitative Insulin Sensitivity Check Index; TG/HDL, Triglycerides HDL-cholesterol index, Data are expressed as mean (SD) or median (interquartile range). Student t or *Wilcoxon test was used.

On multiple linear regression analyses, associations of LBW with markers of beta-cell function and insulin sensitivity were tested. LBW was associated with log-transformed HOMA-β values (p = 0.014), but not with the other indexes of insulin sensitivity (**Table 2**).

**Table 2:** Association of low birth weight with parameters of β-cell function and insulin sensitivity.

	β	95% CI	*P-*value
**HOMA-β^#^**			
Model 1	−0.03	−0.054–0.003	0.080
Model 2	−0.03	−0.054–0.003	0.082
Model 3	−0.04	−0.072–0.008	0.014
**HOMA-IR^#^**			
Model 1	−0.02	−0.051–0.011	0.198
Model 2	−0.02	−0.050–0.012	0.233
Model 3	−0.03	−0.065–0.005	0.089
**HOMA-AD^#^**			
Model 1	0.000	−0.110–0.110	0.997
Model 2	0.006	−0.106–0.117	0.922
Model 3	0.027	−0.100–0.155	0.667
**TyG**			
Model 1	0.002	−0.013–0.017	0.754
Model 2	0.004	−0.012–0.019	0.636
Model 3	−0.001	−0.018–0.016	0.899
**QUICKI^#^**			
Model 1	0.003	−0.001–0.008	0.184
Model 2	0.003	−0.002–0.007	0.215
Model 3	0.005	−0.000–0.009	0.077
**TG/HDL^#^**			
Model 1	−0.001	−0.036–0.033	0.945
Model 2	0.002	−0.033–0.037	0.898
Model 3	−0.007	−0.045–0.032	0.725

Model 1: adjusted for sex and skin color.

Model 2: adjusted for sex, skin color and family history of diabetes.

Model 3: adjusted for sex, skin color, family history of diabetes and prematurity.

^#^Log-transformed values of outcomes for analyses.

### Propensity-Score Matching—Variable Balance

Initially, the sample contained 238 participants with LBW. After applying the propensity-score matching, the final samples included 227 matched participants for HOMA-β, HOMA-IR, TyG, QUICKI and TG/HDL analysis and 64 matched participants for HOMA-AD analysis. The matching approach made all covariates appropriately balanced (standardized mean difference of between −0.1 and 0.1) for further analyses. Variable balance was compared before and after matching to assess the improvement of pairing (**Supplementary Table S1**).

#### Associations of LBW With β-Cell Function

After propensity-score matching, the multiple linear regression model, adjusted for sex, skin color, family history of diabetes and prematurity, showed no association between LBW and HOMA-β (ß 0.003, 95%CI −0.038–0.045 p = 0.107) (**Table 3**).

**Table 3:** Estimates of associations of LBW with parameters of β-cell function and insulin sensitivity after propensity-score matching in ELSA-Brasil participants.

	Propensity-score pairing		
	Coefficient (ß)	95% CI	*P*-value
HOMA-β^#^	0.003	−0.038–0.045	0.107
HOMA-IR^#^	0.0004	−0.00037–0.003	0.818
HOMA-AD^#^	0.046	0.015–0.078	0.005
TyG	0.009	−0.017–0.018	0.991
QUICKI^#^	0.0008	−0.0009–0.0092	0.986
TG/HDL^#^	0.021	0.013–0.036	<0.001

HOMA-IR, Homeostasis Model Assessment of Insulin Resistance; AD, Adiponectin; QUICKI, Quantitative Insulin Sensitivity Check Index; TG/HDL, Triglycerides HDL-c; CI, confidence interval.

^#^Log-transformed values for analyses.

#### Associations of LBW With Insulin Sensitivity

The fully adjusted multiple linear regression model showed that being born with LBW was directly associated with HOMA-AD (ß 0.046, 95% CI 0.015–0.078, p = 0.005) and TG/HDL index (ß 0.021, 95% CI 0.013–0.036, p <0.001). There was no association of LBW with HOMA-IR, TyG or QUICKI (**Table 3**).

## Discussion

We found evidence further supporting the hypothesis that LBW is associated with decreased β-cell function and with insulin resistance in middle-aged non-diabetic participants from the ELSA-Brasil (18, 19). The study findings are strengthened by the facts that several indexes of insulin secretion and sensitivity were used and DAG applied for adjustments and propensity-score matching analysis. An association was found of LBW with HOMA-AD and TG/HDL indexes, after adjustment and propensity-score matching. No association between low birth weight and HOMA-β was found. The results reinforced the possible role of early life events in insulin sensitivity, even with marker values within the normal range in adults born with LBW.

Considering the magnitude of T2DM as a public health concern, causing morbidity and mortality worldwide (29, 30), initiatives to improve prediction and prevention are timely. The present study was prompted by evidence that population-attributable risk of T2DM is associated with increased mortality in adults born with LBW compared to those with normal birth weight (16). Additionally, LBW has been associated with hyperinsulinemia and increased risk of diabetes later in childhood (31, 32). We hypothesized that these abnormalities can affect pancreatic function during the life course, justifying the assessment of beta cell secretion capacity, and peripheral insulin sensitivity before glucose metabolism disturbances emerged. In this context, our study evaluated traditional and novel indexes of β-cell function and insulin sensitivity/resistance.

HOMA-β and HOMA-IR are the most common indexes for estimating insulin secretion and resistance (33). In the present study, HOMA-AD was also calculated to assess insulin sensitivity. This index is a modified version of HOMA-β which incorporates the total serum adiponectin level in the denominator to indirectly adjust to degree of body adiposity. Adiponectin is a protein involved in the pathophysiology of obesity and low levels tend to be observed in obese individuals with ectopic adipose tissue deposition (34). Hypoadiponectinemia has been considered an independent risk factor for the development of T2DM (35). To the best of our knowledge, the present study is the first to assess insulin sensitivity in overweight adults with LBW. HOMA-AD has been evaluated in the pediatric population, individuals with chronic kidney disease and chronic liver disease (36–38). We also calculated TG/HDL ratio, an alternative, low-cost, useful index for clinical practice. These lipid parameters are typically altered in individuals with the metabolic syndrome, in which insulin resistance is the main pathophysiological event. Both HOMA-AD and TG/HDL were associated with LBW in the well-balanced sample after applying propensity-score matching. Considering that most people are born with normal birth weight, as was the case in the present sample, this analysis was valid for improving the reliability of comparisons of subgroups stratified according to birth weight. Several studies have shown that HOMA-AD offers greater accuracy than HOMA-IR for assessing insulin resistance in overweight non-diabetic individuals (39, 40). Given that diabetic individuals were excluded from the study sample, the findings regarding the HOMA-AD and TG/HDL indexes suggest their utility for early detection of insulin resistance in middle-aged adults.

In the natural history of T2DM, insulin resistance precedes the decline of β cell function and is associated with ectopic fat deposition in the liver, muscles and pancreas (41). In turn, weight loss can prevent this condition by improving insulin sensitivity. Our results are consistent with insulin resistance preceding β-cell dysfunction in overweight adults who have not developed a glucose metabolism disturbance. LBW was initially associated with HOMA-β on multiple linear regression. However, after applying propensity-score matching in a well-balanced sample, including for the adiposity parameter (BMI), this association no longer persisted. Therefore, these results revealed an association of LBW with insulin sensitivity markers in middle-aged adults, where an association with HOMA-β can be expected in the long term if a weight loss intervention is not pursued. To our knowledge, no previous studies have reported the use of HOMA-AD and TG/HDL indexes as early markers of insulin sensitivity in adults who still have preserved β-cell function.

Explanations for these findings are based on the reported associations of LBW and glucose metabolism dysfunction, and particularly when these infants also experience catch-up growth in childhood. These individuals are prone to developing obesity, increased visceral adiposity and insulin resistance (42, 43). An elevated number of insulin receptors in their adipocytes and abnormal signaling by phosphorylation of insulin-receptor substrate 1 may result in an anti-lipolysis state (44, 45). Also, it has been shown that each tertile decrease in birth weight was associated with a 1.72 times greater risk of insulin resistance in adults (46). Concordantly, our data favor the hypothesis that once insulin resistance is installed, insulin production will increase and can progress to β-cell failure over time. Other studies support the possibility that decreased β-*cell function can occur without insulin resistance. This was observed in individuals with intrauterine growth restriction who had a marked reduction in number of β cells (47–49). Another study confirmed that adults born with LBW had a 30% reduction in insulin secretion (10). Animal models involving intrauterine energy restriction showed similar results, with a reduction in β cell mass of up to 35% (48). Despite uncertainties over the underlying mechanisms, the present results support the occurrence of early onset of insulin resistance in adults without diabetes.

Other indexes could have been useful to assess β cell function such as the OGIS (50) and Matsuda index (51) that require several determinations of plasma glucose and insulin during glucose tolerance tests. More recently, insulin clearance was raised as an important aspect of glucose metabolism and its impairment has been related to the risk of developing T2DM (52–54). Although this method would enhance the β cell function evaluation, measurements for its estimation were not available in the ELSA-Brasil.

The present study has limitations related to recall bias, given that retrospective data were collected regarding early life events. This bias can be reduced by using a sample of middle-aged participants, under 60 years of age. Some studies have shown that perinatal-related events are reliably reported during adult life (55–57). We also use the exposure (LBW) as a categorical variable, possibly reducing the statistical power of the analysis. This approach was chosen to minimize information inaccuracy from participants who were unable to accurately recall their birth weight in kilograms. The use of the propensity-score method decreased the sample size, limiting the ability to find valid associations. Therefore, future studies investigating the association between LBW and HOMA-β and other indexes in larger samples are needed. A cross-sectional analysis of the ELSA-Brasil data was conducted. Further analyses of the follow-up of the sample can allow causality between LBW and the occurrence of glucose metabolism disturbances to be explored.

A strength of this study was the methodological approach employed, including the Directed Acyclic Graph method to identify confounding variables, avoiding over adjustments in the regression models constructed (25, 26). Although a variety of covariates were controlled for, other exposures which occurred during the life course of participants were not included. However, we collected body weight at age 20 in an attempt to define participant body weight trajectory. Another strength was the use of propensity-score matching to reduce potential selection confounders seen in observational studies (27, 28), achieving sufficiently balanced groups in the analysis. Furthermore, the frequency of self-reported LBW found in the sample was comparable to that reported in the Brazilian population at large (58).

In conclusion, LBW was found to be associated with insulin sensitivity markers in adulthood before overt glucose metabolism disturbances emerged. HOMA-AD and TG/HDL indexes appeared to be useful for detecting insulin resistance in overweight adults who had LBW. These findings are relevant in reinforcing the hypothesis that early life events affect glucose metabolism during the life course. Thus, identifying the subset of individuals at risk may be important to allow early implementation of preventive measures.

## Data Availability Statement

The original contributions presented in the study are included in the article/**Supplementary Material**. Further inquiries can be directed to the corresponding author.

## Ethics Statement

The study was approved by the National Commission on Ethics Research (CONEP) and the local ethics committee, the Research Ethics Committee (CEP) under registration number 76 of the University of São Paulo (HU-USP). The patients/participants provided their written informed consent to participate in this study.

## Author Contributions

Study design, analysis, interpretation and preparation of the manuscript: JB, BA-P and SF. Acquisition of data and critical revision for the manuscript content: PL and IB. All authors listed have made a substantial, direct, and intellectual contribution to the work and approved it for publication.

## Funding

The current study was supported by a grant from the São Paulo Research Foundation (Fundação de Amparo à Pesquisa do Estado de São Paulo—FAPESP—Protocol 2009/15041-9). Also it was financed in part by the Coordenação de Aperfeiçoamento de Pessoal de Nível Superior—Brasil (CAPES)—(Finance Code 001). The baseline ELSA-Brasil study was supported by the Brazilian Ministry of Health (Science and Technology Department), the Brazilian Ministry of Science and Technology, and CNPq-National Research Council (# 01 06 0010.00 RS, 01 06 0212.00 BA, 01 06 0300.00 ES, 01 06 0278.00 MG, 01 06 0115.00 SP, 01 06 0071.00 RJ).

## Conflict of Interest

The authors declare that the research was conducted in the absence of any commercial or financial relationships that could be construed as a potential conflict of interest.

## Publisher’s Note

All claims expressed in this article are solely those of the authors and do not necessarily represent those of their affiliated organizations, or those of the publisher, the editors and the reviewers. Any product that may be evaluated in this article, or claim that may be made by its manufacturer, is not guaranteed or endorsed by the publisher.
