# Supplementary material for: Low Birth Weight, β-Cell Function and Insulin Resistance in Adults: The Brazilian Longitudinal Study of Adult Health
Source: Front Endocrinol (Lausanne). 2022 Mar 14;13:842233. doi: 10.3389/fendo.2022.842233 (PMC8964259; doi:10.3389/fendo.2022.842233)
Supplement: Supplementary file 1 [file DataSheet_1.pdf]

SUPPLEMENTARY MATERIAL

Figure S1. Directed acyclic graph for β-cell function (panel A) and Insulin Sensitivity (panel B).

A

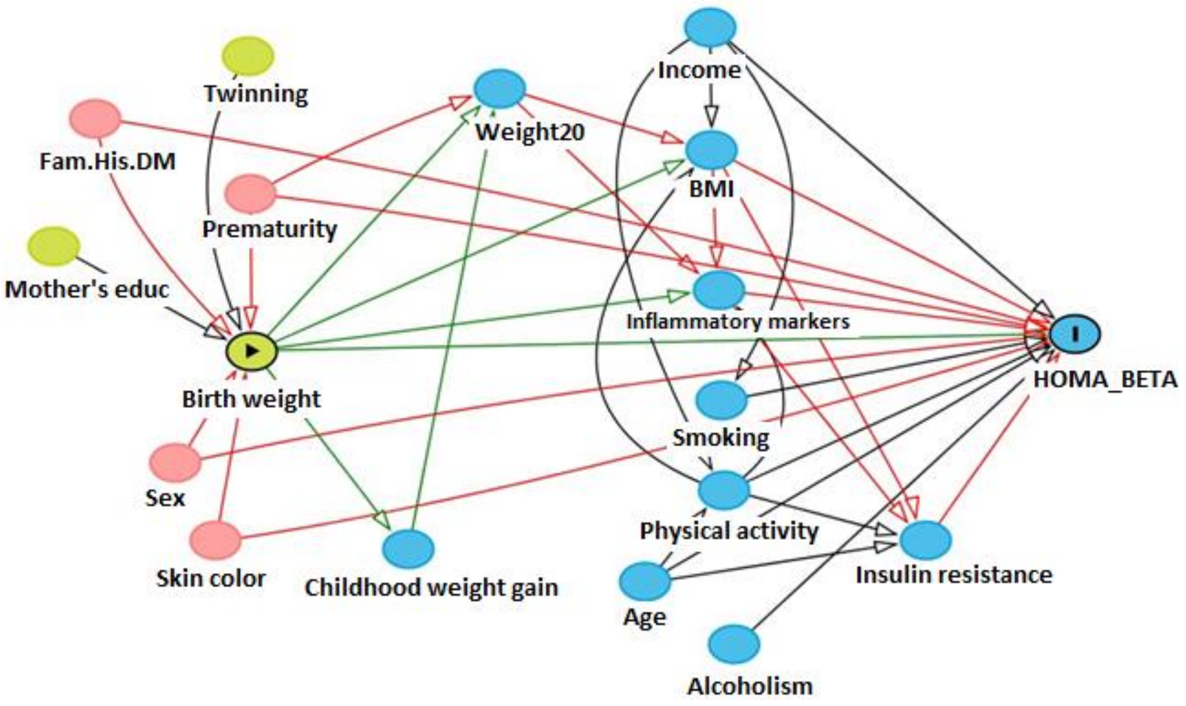

B

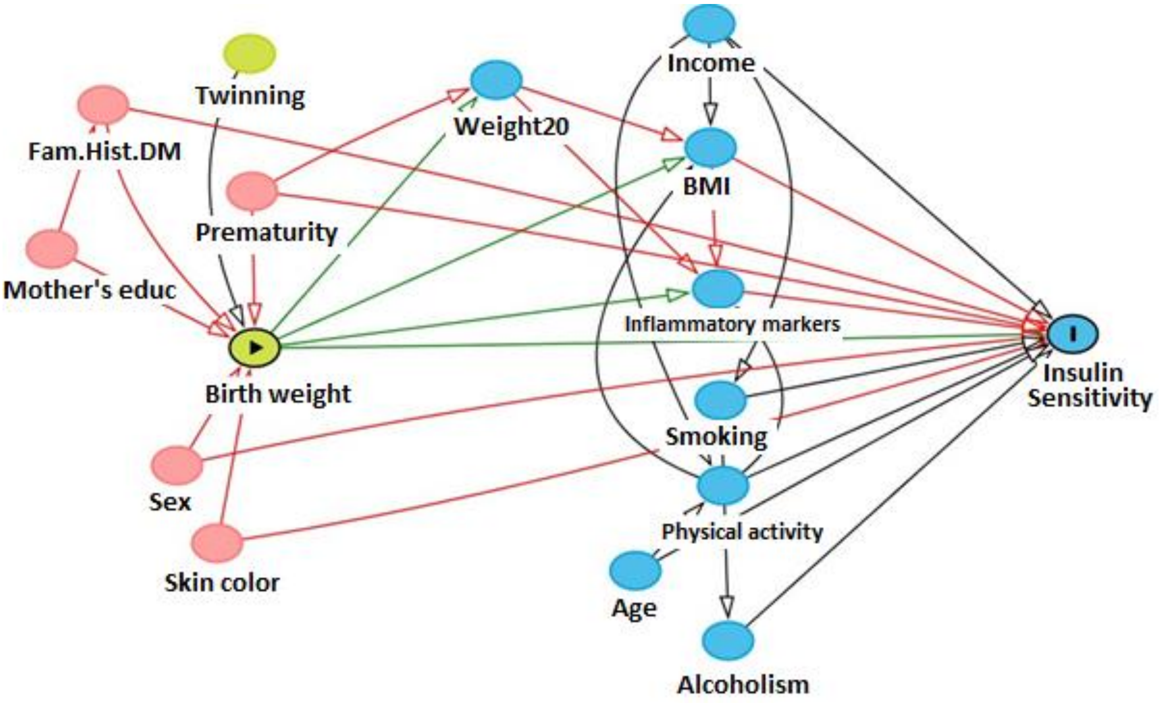

Table S1. Results of propensity-score matching: standardized mean differences of adjusted variables for association of LBW with β-cell function and insulin sensitivity markers of ELSA-Brasil participants.

Variables	Summary of balance		
	**Crude**	**Balance for matched data**	**% Balance improvement**
HOMA-β# (n = 227)			
Skin color	-0.221	-0.070	68.48
Prematurity	0.699	0.000	100.0
Sex	-0.118	-0.051	56.27
Family history of diabetes	-0.106	-0.021	80.41
HOMA-IR# (n = 227)			
Skin color	-0.221	-0.040	81.81
Prematurity	0.699	0.000	100.0
Sex	-0.118	-0.073	38.16
Family history of diabetes	-0.106	0.032	70.32
HOMA-AD# (n = 64)			
Skin color	-0.101	0.160	-58.26
Prematurity	0.757	0.000	100.0
Sex	-0.005	-0.080	-146.0
Family history of diabetes	-0.277	-0.177	36.22
TyG (n = 227)			
Skin color	-0.221	0.000	100.0
Prematurity	0.699	0.000	100.0
Sex	-0.118	-0.102	12.97
Family history of diabetes	-0.106	-0.010	90.25
QUICKI# (n = 227)			
Skin color	-0.221	0.040	81.99
Prematurity	0.699	0.000	100.0
Sex	-0.118	-0.010	91.25
Family history of diabetes	-0.106	-0.031	70.61
TG/HDL# (n = 227)			
Skin color	-0.221	0.000	100.0
Prematurity	0.699	0.000	100.0
Sex	-0.118	-0.103	12.54

Family history of diabetes	-0.106	-0.042	60.82

HOMA-IR, Homeostasis Model Assessment of Insulin Resistance; AD, Adiponectin; QUICKI, Quantitative Insulin Sensitivity Check Index; TG/HDL, Triglycerides HDL-cholesterol index. CI, confidence interval.

Log-transformed values for analyses
